# Parathyroid hormone alleviates non-alcoholic liver steatosis *via* activating the hepatic cAMP/PKA/CREB pathway

**DOI:** 10.3389/fendo.2022.899731

**Published:** 2022-08-17

**Authors:** Xu Feng, Ye Xiao, Qi Guo, Hui Peng, Hai-Yan Zhou, Jian-Ping Wang, Zhu-Ying Xia

**Affiliations:** ^1^ Department of Endocrinology, Endocrinology Research Center, Xiangya Hospital of Central South University, Changsha, China; ^2^ Department of Endocrinology, The Second Affiliated Hospital of University of South China, Hengyang, China; ^3^ National Clinical Research Center for Geriatric Disorders, Xiangya Hospital, Changsha, China

**Keywords:** parathyroid hormone, non-alcoholic liver steatosis, therapeutics, PTH1R, PKA/CREB pathway

## Abstract

Non-alcoholic fatty liver disease (NAFLD), hallmarked by liver steatosis, is becoming a global concern, but effective and safe drugs for NAFLD are still lacking at present. Parathyroid hormone (PTH), the only FDA-approved anabolic treatment for osteoporosis, is important in calcium-phosphate homeostasis. However, little is known about its potential therapeutic effects on other diseases. Here, we report that intermittent administration of PTH ameliorated non-alcoholic liver steatosis in diet-induced obese (DIO) mice and db/db mice, as well as fasting-induced hepatic steatosis. *In vitro*, PTH inhibits palmitic acid-induced intracellular lipid accumulation in a parathyroid hormone 1 receptor (PTH1R)-dependent manner. Mechanistically, PTH upregulates the expression of genes involved in lipid β-oxidation and suppresses the expression of genes related to lipid uptake and *de novo* lipogenesis by activating the cAMP/PKA/CREB pathway. Taken together, our current finding proposes a new therapeutic role of PTH on NAFLD.

## Introduction

Non-alcoholic fatty liver disease (NAFLD) is hallmarked by massive macrovesicular and/or microvesicular steatosis in the liver in the absence of heavy drinking (1). In recent years, the prevalence of the non-alcoholic fatty liver disease is increasing globally, and it is set out to be the predominant cause of chronic liver disease worldwide (2, 3). However, effective and safe drugs for NAFLD are lacking at present (4–7). Thus, searching for agents efficiently targeting NAFLD is highly desirable.

Parathyroid hormone (PTH) is a hormone secreted by the parathyroid glands, which is important in regulating serum calcium-phosphate homeostasis and bone remodeling (8). Besides its classical actions on bone and kidney, accumulating evidence indicated that PTH may have other important metabolic effects. In previous studies, PTH has been proved to act on adipose tissues and bone marrow to induce lipolysis or browning of adipocytes (9–14). Recently, Sachiko et al. demonstrated that PTH administration benefits hepatic lipid metabolism in a rat model of type 2 diabetes mellitus (15). However, whether PTH has a pervasive effect on non-alcoholic liver steatosis and the underlying mechanisms for PTH regulating hepatic lipid metabolism is not clear.

PTH (1–34)(hereinafter referred to as PTH), the N-terminal fragment of the intact hormone, is the only FDA-approved anabolic agent capable to stimulate bone formation (16). In this study, we show that intermittently subcutaneous injection of PTH (40 μg/kg daily) could alleviate the non-alcoholic liver steatosis in diet-induced obese (DIO) mice and leptin receptor-deficient (db/db) mice (a classic animal model for liver steatosis) (17), and reduce the hepatic lipid accumulation in 24-hour-fasted mice. Furthermore, siRNA-mediated parathyroid hormone 1 receptor (PTH1R) knockdown significantly reversed the inhibitory effects of PTH on palmitic acid (PA)-induced lipid accumulation in Hepa1-6 cells. Mechanistically, PTH upregulates the expression of genes related to lipid β-oxidation and suppresses the expression of genes involved in *de novo* lipogenesis and lipid uptake *via* activating the cAMP/PKA/CREB pathway. Together, our study suggests a new therapeutic role of PTH on NAFLD.

## Materials and methods

### Animals

Male C57BL/6J mice and db/db mice were purchased from Hunan SJA Laboratory Animal Company. All animals used in this study were maintained in the specific pathogen-free animal facility of the Laboratory Animal Research Center of Central South University with ad libitum access to a normal chow diet (NCD) and water. For the DIO model, eight-week-old male C57BL/6J mice were fed with a 60 kcal% high-fat diet (HFD, D12492; Research Diets) for 16 weeks (18, 19). For the fasting model, food was removed before lights off for 24h (20, 21). For PTH treatment, mice were subcutaneously injected with PTH peptide (synthesized by KE Biochem Co. Ltd) at the dose of 40 μg/kg/day during the experiment (22–26), and the control group was injected with an equal volume of PBS. All animal experimental procedures and protocols were reviewed and approved by the Laboratory Animal Ethics Committee at Central South University (2020SYDW0613), and this study was compliant with all relevant ethical regulations regarding animal research.

### Insulin tolerance test (ITT)

The insulin tolerance tests (ITTs) were performed as previously reported (27, 28). Mice were fasted for 4h, then intraperitoneally injected with 0.75U/kg insulin. Tail venous blood was collected at 0, 15, 30, 60, 90, and 120 min after insulin injection and measured by glucometer to determine the blood glucose levels at different time 1points.

### Serum analysis and hepatic lipid measurement

Serum samples and hepatic lipids were collected and extracted as previously reported (29). Total glyceride (TG), total cholesterol (TC), alanine transaminase (ALT), and aspartate aminotransferase (AST) levels were measured with the corresponding kit respectively, according to the manufacturers’ instructions. All these kits were purchased from Shanghai Shensuo UNF Medical Diagnostic Articles Company (Shanghai, China).

### Cell culture and treatment

Hepa1-6 cells were purchased from Procell Life Science & Technology Co., Ltd. (Wuhan, China), and cultured in DMEM(Gibco) supplemented with 10% (v/v) FBS(Gibco) plus 50 U/ml penicillin and 50 μg/ml streptomycin (Solarbio) at 37°C in a humidified incubator with 5% CO_2_. PTH1R-targeted small interfering RNAs (siRNAs) were purchased from Ribobio (Guangzhou, China) and transfected using lipofectamine 2000 (Invitrogen) following the instructions. qPCR and western blot were used to determine the knockdown efficiency 2 days after transfection. For assessment of cellular signal pathway, cells were treated with 10 nM PTH for 1h with or without preincubation of 10 μM H89 (Selleck) for 1h (12). For assessment of downstream gene expression, cells were treated with 10 nM PTH for 24h with or without preincubation of H89.

### Induction and measurement of steatosis *in vitro*

*In vitro* hepatic steatosis was induced as previously described with a slight modification (30). The palmitic acid solvent (6mM, Sigma) was prepared in 20% fatty acid-free BSA. Hepa1-6 cells were treated with 0.3 mM PA or 1% BSA for 24h in 12-well plates. Cells were stained with Oil Red O to examine the amount of lipid accumulation in the cells. Three images from each well were photographed, and a representative image is shown. For quantitative assessment, 100% 2-propanol was added to the plates and the absorption of the elution was measured in duplicate at 510 nm (31).

### qPCR

Total RNAs of tissue or cultured cells were extracted using AG RNAex Pro Reagent (Accurate Biotechnology (Hunan) Co., Ltd). Then, cDNA synthesis and qPCR assay were performed as previously reported (32, 33). The primer pairs used for qPCR were listed in **Supplemental Table 1**. The relative mRNA levels were calculated by the 2^−ΔΔCt^ method using Gapdh as an internal control.

### Western blotting analysis

Western blot analysis was performed as previously reported (34, 35). The primary antibodies used in Western blot analysis are mouse anti–PGC-1α (Santa Cruz, sc-517380), mouse anti-PPARγ (Santa Cruz, sc-7273), mouse anti-PTH1R (Santa Cruz, sc-12722), mouse anti–GAPDH (Origene, TA802519), rabbit anti- Phospho-(Ser/Thr) PKA Substrate (Cell Signaling Technology, 9621), rabbit anti- Phospho-CREB (Ser133) (Cell Signaling Technology, 9198), rabbit anti-CREB (Proteintech, 12208-1-AP).

### Histological analysis of tissues

Histological analysis was performed as previously reported (36). The liver samples were fixed in 4% paraformaldehyde, embedded in paraffin, sectioned, and stained with H&E for histology. To determine hepatic lipid accumulation, paraformaldehyde-fixed liver samples were dehydrated in sucrose, embedded in OCT, and frozen sections of the liver were stained with Oil Red O.

### Statistics

Data are presented as the means ± SD from at least four animals or three independent experiments, and representative results are shown. For comparisons of two groups, a two-tailed Student’s t-test was used. A one- or two-way ANOVA was performed for comparisons of multiple groups. Differences were considered significant at P value less than 0.05 (*P < 0.05; **P < 0.01; ***P < 0.001; ****P < 0.0001).

## Results

### Intermittent administration of PTH reduces body weight and fat mass in DIO and db/db mice

**Figure 1:**
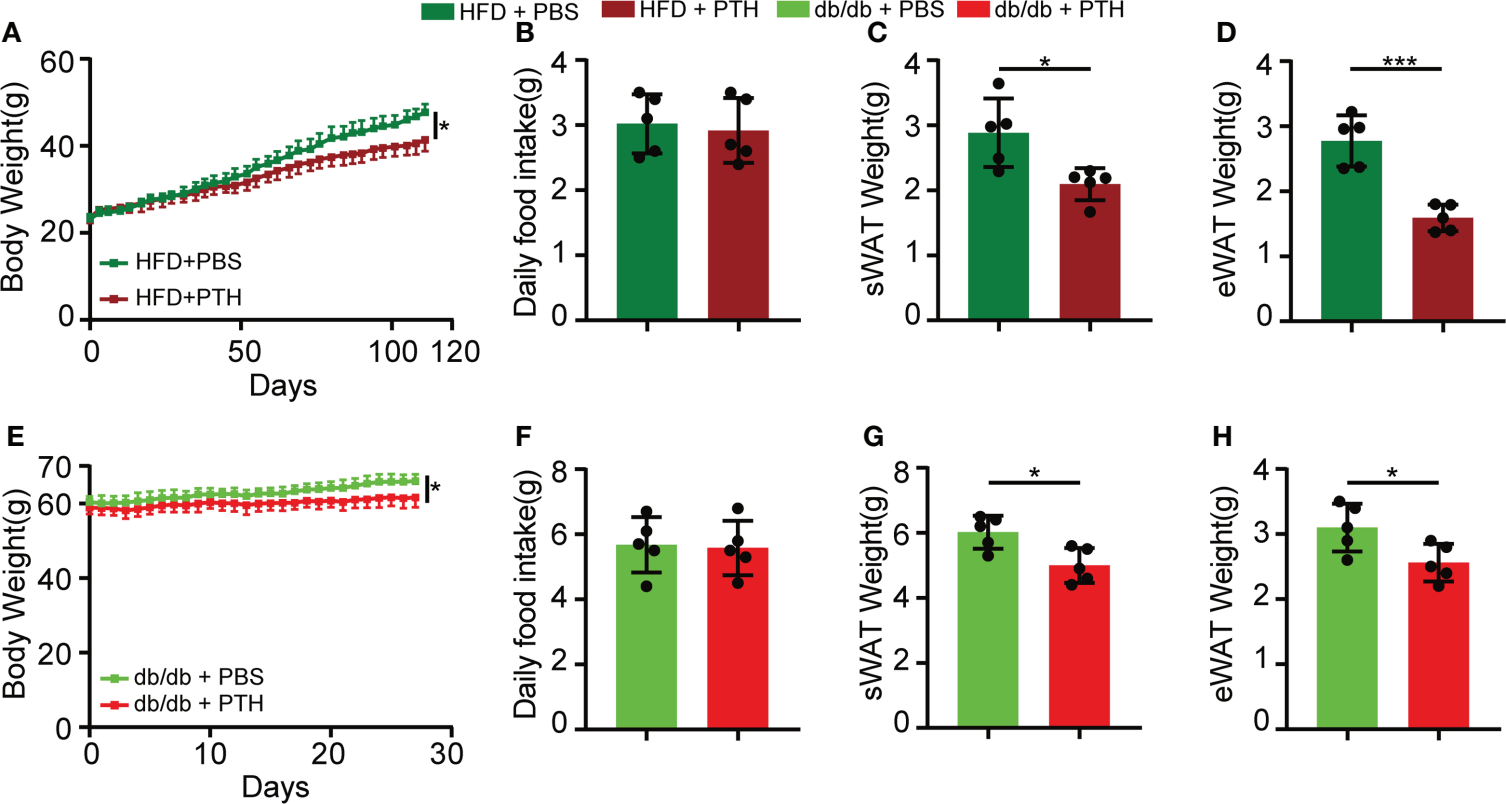
Intermittent administration of PTH reduced body weight and fat mass in DIO mice and db/db mice. **(A–D)** Eight-week-old C57/BL6 male mice were fed on a high-fat diet (HFD) for 16 weeks. During the period of HFD feeding, the mice were subcutaneously injected with PTH (40 μg/kg daily) or PBS. **(A)** Body weight curve. **(B)** Daily food intake. **(C, D)** The weights of sWAT and eWAT in DIO mice. **(E–H)** Twenty-week-old male db/db mice were subcutaneously injected with PTH (40 μg/kg daily) or PBS for 1 month. **(E)** Body weight curve. **(F)** Daily food intake. **(G, H)** The weights of sWAT and eWAT in db/db mice. Data are shown as mean ± SD (n=5/group). *p < 0.05; ***p < 0.001 by two-tailed Student’s t-test or two-way ANOVA.

We first examined whether exogenous PTH affects the development of obesity. To minimize the side effects of PTH, we chose the commonly used subcutaneous dose of PTH at 40μg/kg by intermittent administration in this study (22–26). Eight-week-old C57/BL6 mice were fed with HFD for 16 weeks as DIO mice. During the sixteen-week feeding, mice were treated with intermittently subcutaneous injection of PTH (40 μg/kg daily) or PBS as a control. PTH treatment slightly decreased the body weight of DIO mice (**Figure 1A** and **Supplementary Figure 1A**) and did not affect the daily food intake (**Figure 1B**), indicating a potential prophylactic effect of PTH on diet-induced obesity. In accordance, the weights of subcutaneous white adipose tissue (sWAT, **Figure 1C** and **Supplementary Figure 1B**) and epididymal white adipose tissue (eWAT, **Figure 1D** and **Supplementary Figure 1B**) significantly decreased in DIO mice treated with PTH, partially contributing to the decrease in body weight. To investigate the therapeutic effect of PTH on obese mice at mid-life, twenty-week-old male db/db mice were subcutaneously injected with PTH (40 μg/kg daily) or PBS for 1 month. At the termination of treatment, PTH-treated db/db mice showed a slightly but significantly decrease in the body weight and weights of sWAT and eWAT, while food intake did not appear to be affected either (**Figures 1E** **–H**). Taken together, these results indicated the potential prophylactic and therapeutic effects of PTH in obese mice.

### PTH alleviates hepatic steatosis in DIO mice and db/db mice

**Figure 2:**
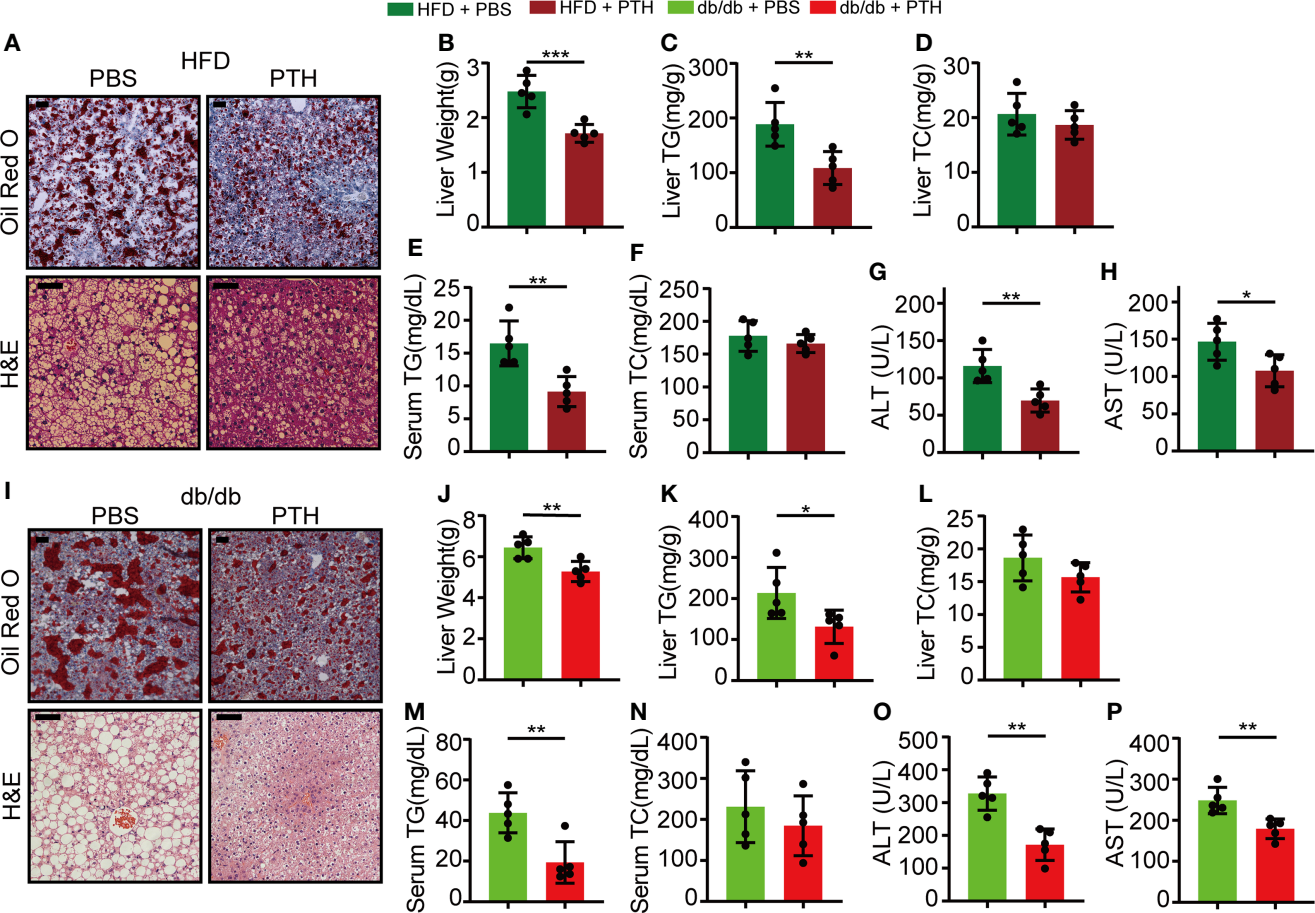
PTH alleviated hepatic steatosis in DIO mice and db/db mice. **(A)** The Oil Red O and H&E staining of liver tissues in DIO mice (Scale bar = 100 μm). **(B)** Liver weight. **(C, D)**The hepatic TG and TC levels. **(E, F)** The serum TG and TC levels. **(G, H)** The serum ALT and AST levels. **(I)** The Oil Red O and H&E staining of liver tissues in db/db mice (Scale = bar 100 μm). **(J)** Liver weight. **(K, L)** The hepatic TG and TC levels. **(M, N)** The serum TG and TC levels. **(O, P)** The serum ALT and AST levels. Data are shown as mean ± SD (n=5/group). *p < 0.05; **p < 0.01; ***p < 0.001 by two-tailed Student’s t-test.

We next focused on the role of PTH in the liver. Non-alcoholic liver steatosis results from abnormal lipid metabolism in the liver and eventually leads to liver injury (1, 4, 7). The hepatic fat vacuoles and accumulated lipid droplets in DIO mice shown by H&E and Oil Red O staining were reversed by PTH treatment (**Figure 2A**). The liver weights of the PTH-treated DIO mice were significantly lower compared to the control group (**Figure 2B**). Consistently, the TG levels in the liver and serum significantly decreased in PTH-treated DIO mice (**Figures 2C, E**), while there was no significant difference in the liver and serum TC levels (**Figures 2D** **, F**). Furthermore, PTH treatment decreased serum levels of ALT and AST reflecting improved liver function (**Figures 2G, H**). In parallel, similar phenotypes were observed in PTH-treated db/db mice. PTH-treated db/db mice showed decreased hepatic fat vacuoles and lipid droplets (**Figure 2I**), decreased liver weight (**Figure 2J**) and decreased TG levels in liver and serum (**Figures 2K, M**). The TC levels in the liver and serum showed no significant difference (**Figures 2L, N**). Decreased serum levels of ALT and AST indicated ameliorated liver injury in PTH-treated db/db mice (**Figures 2O, P**). These findings showed that PTH could ameliorate hepatic steatosis in obese mice.

### PTH ameliorates fasting-induced hepatic steatosis

**Figure 3:**
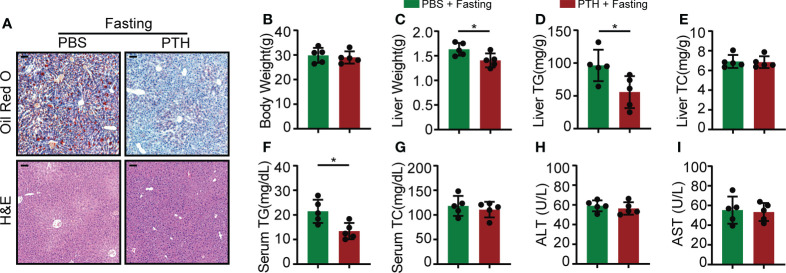
The fasting-induced lipid accumulation in the liver of mice was ameliorated by PTH administration. Eight-week-old male C57/BL6 mice were subcutaneously injected with PTH (40 μg/kg daily) or PBS for 2 months, then fasted for 24 hours and sacrificed. **(A)** The Oil Red O and H&E staining of mice liver tissues (Scale bar = 100 μm). **(B)** Body weight. **(C)** Liver weight. **(D, E)** The hepatic TG and TC levels. **(F, G)** The serum TG and TC levels. **(H, I)** The serum ALT and AST levels. Data are shown as mean ± SD (n=5/group). *P < 0.05 by two-tailed Student’s t-test.

Given the involvement of PTH in adipocyte lipolysis (11–14), we thought to determine whether the lipolytic action could lead to hepatic steatosis under NCD feeding. Eight-week-old C57/BL6 mice were subcutaneously injected with PTH (40 μg/kg daily) or PBS for 2 months and fed with NCD. There was no difference in body weight gain, daily food intake, and liver weight between the PTH and control group (**Supplementary Figures 2A–C**). H&E staining showed no apparent histological changes in the liver of PTH-treated mice compared with the control group (**Supplementary Figure 2D**). These data suggested that PTH treatment at least at the present dose did not disturb body weight or lead to hepatic steatosis under NCD feeding. We next investigated whether PTH affects acute fasting-induced steatosis, which is attributed to excess serum free fatty acid released by adipocytes (37). Acute fasting induced large amounts of microscopically visible lipid droplets accumulated in the liver (**Supplementary Figure 3A**) and decreased both body weight and liver weight (**Supplementary Figures 3B, C**). Acute fasting drastically increased the TG levels in the liver, while hepatic TC, serum TG, TC, ALT, and AST levels were not changed (**Supplementary Figures 3D–I**). Of interest, PTH treatment protected against fasting-induced accumulation of lipid droplets as displayed by H&E and Oil O Red staining (**Figure 3A**). While the two groups showed similar body weights after fasting, the liver weights of PTH-treated mice were lower than that of PBS-treated mice (**Figure 3B**). Consistent with these observations, PTH treatment decreased both hepatic and serum TG levels (**Figures 3D, F**). As for hepatic TC (**Figure 3E**), serum TC, ALT, and AST (**Figures 3G** **–I**), there was not much difference between the two groups. These data showed that intermittent administration of PTH could alleviate fasting-induced hepatic steatosis without affecting body weight under NCD feeding, indicating a direct action of PTH on hepatic lipid metabolism.

### PTH promotes hepatic lipid metabolism in obese mice

**Figure 4:**
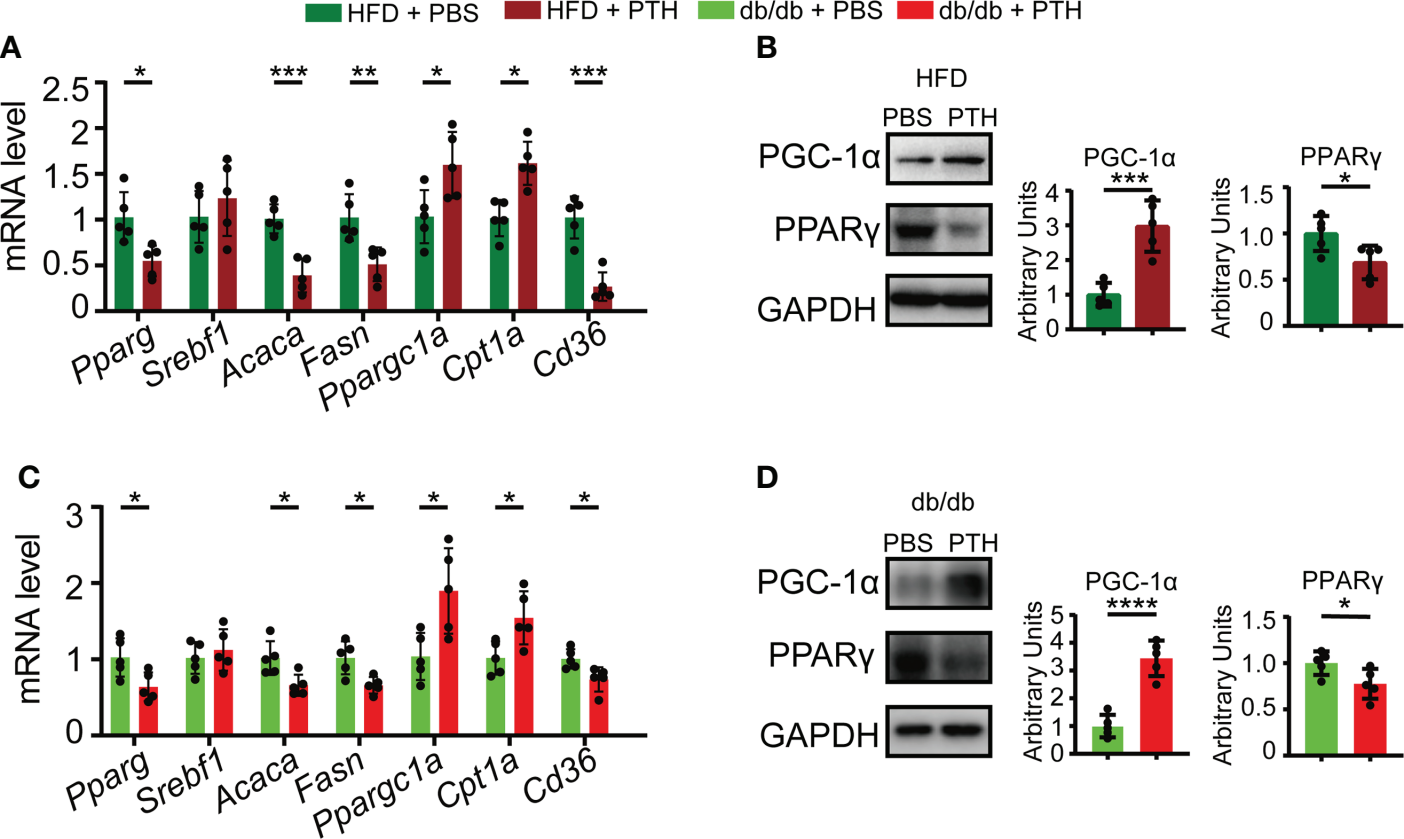
Effects of PTH on hepatic lipid metabolism-related genes. **(A)** The mRNA levels of genes involved in *de novo* lipogenesis, β-oxidation, and uptake of lipid in the liver of DIO mice injected with PTH or PBS as indicated. **(B)** The protein levels of PGC-1α and PPARγ in the liver of DIO mice injected with PTH or PBS as indicated. **(C)** The mRNA levels of genes involved in *de novo* lipogenesis, β-oxidation, and uptake of lipid in the liver of db/db mice injected with PTH or PBS as indicated. **(D)** The protein levels of PGC-1α and PPARγ in the liver of db/db mice injected with PTH or PBS as indicated. Data are shown as mean ± SD (n=5/group). *p < 0.05; **p < 0.01; ***p < 0.001; ****p < 0.0001 by two-tailed Student’s t-test.

We further investigated the molecular changes in the liver of PTH-treated DIO mice and db/db mice. Hepatic lipid metabolism undergoes several important biological processes, including *de novo* lipogenesis, lipid uptake, and β-oxidation of lipid, which are mastered by a set of regulatory factors (1). Our results showed that in DIO mice PTH treatment decreased the expression of lipogenesis genes such as *Pparg*, *Acaca*, and *Fasn*, while the expression of *Srebf1* was not affected. In addition, PTH treatment elevated the expression of lipid β-oxidation-related genes such as *Ppargc1a* and *Cpt1a* and downregulated the expression of *Cd36* for lipid uptake (**Figure 4A**). Correspondingly, the protein level of PGC-1α was increased while PPARγ was decreased by PTH treatment in DIO mice (**Figure 4B**). Parallelly, PTH-treated db/db mice showed a similar expression pattern compared with that of DIO mice. PTH treatment upregulated the expression of *Ppargc1a* and *Cpt1a*, suppressed expression of *Pparg*, *Acaca*, *Fasn*, and *Cd36*, and did not affect the expression of *Srebf1* either (**Figure 4C**). PTH treatment increased the protein level of PGC-1α and decreased the protein level of PPARγ in the liver of db/db mice (**Figure 4D**). These results showed that PTH markedly affected hepatic lipid metabolism by enhancing lipid β-oxidation, suppressing uptake of lipid, and *de novo* lipogenesis in the livers of obese mice.

### PTH1R mediates the effects of PTH on hepatic lipid metabolism

**Figure 5:**
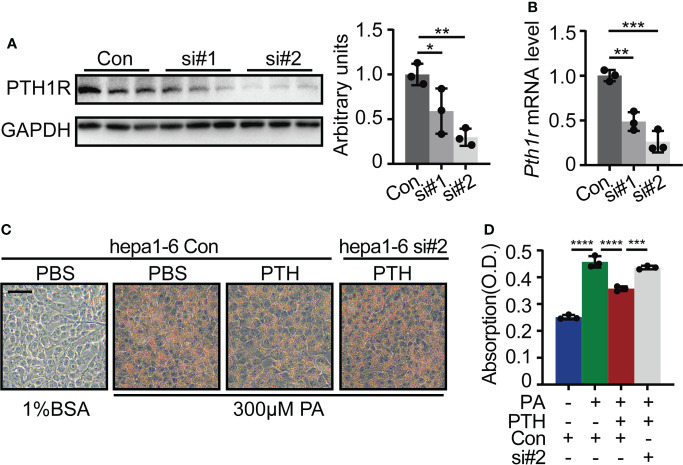
*Pth1r* knockdown abrogated the inhibitory effects of PTH on PA-induced lipid accumulation in Hepa1-6 cells. **(A)** The PTH1R protein level in Hepa1-6 cells transfected with *Pth1r* siRNAs or control. **(B)** The *Pth1r* mRNA level in Hepa1-6 cells transfected with Pth1r siRNAs or control. **(C, D)** Oil Red O staining of Hepa1-6 cells transfected with Pth1r siRNA or control for 30h, and then treated with 0.3 mM PA and 10 nM PTH for 24h **(C)**, Scale bar = 50 μm). Quantitative assessment of intracellular lipid content was determined by the absorption of 2-propanol elution of the cells **(D)**. Data are shown as mean ± SD, the cell experiments were repeated three times. *P < 0.05; **P < 0.01; ***P < 0.001; ****P < 0.0001 by one-way ANOVA.

Previous studies have evidenced the hepatic expression of *Pth1r (* 38, 39), the canonical G protein-coupled receptor for PTH and parathyroid-hormone-related protein (PTHrP) (40). To prove that PTH1R mediated the effects of PTH on lipid accumulation in hepatocytes, we transfected Hepa1-6 cells with *Pth1r* targeted siRNA or siRNA control and then induced intracellular lipid accumulation in Hepa1-6 cells by PA in the presence of PTH. Hepa1-6 cells transfected with *Pth1r* targeted siRNAs showed significantly decreased mRNA and protein levels of *Pth1r* (**Figures 5A, B**). We found that PTH treatment suppressed PA-induced lipid accumulation in the siRNA-control group compared with the siRNA-control plus PBS-treated group (**Figures 5C, D**). However, the inhibitory effects of PTH on PA-induced lipid accumulation were abolished in the siRNA-*Pth1r* group (**Figures 5C, D**). These data suggested that PTH1R mediated the effects of PTH on hepatic lipid metabolism.

### PTH promotes hepatic lipid metabolism through the cAMP/PKA/CREB pathway

**Figure 6:**
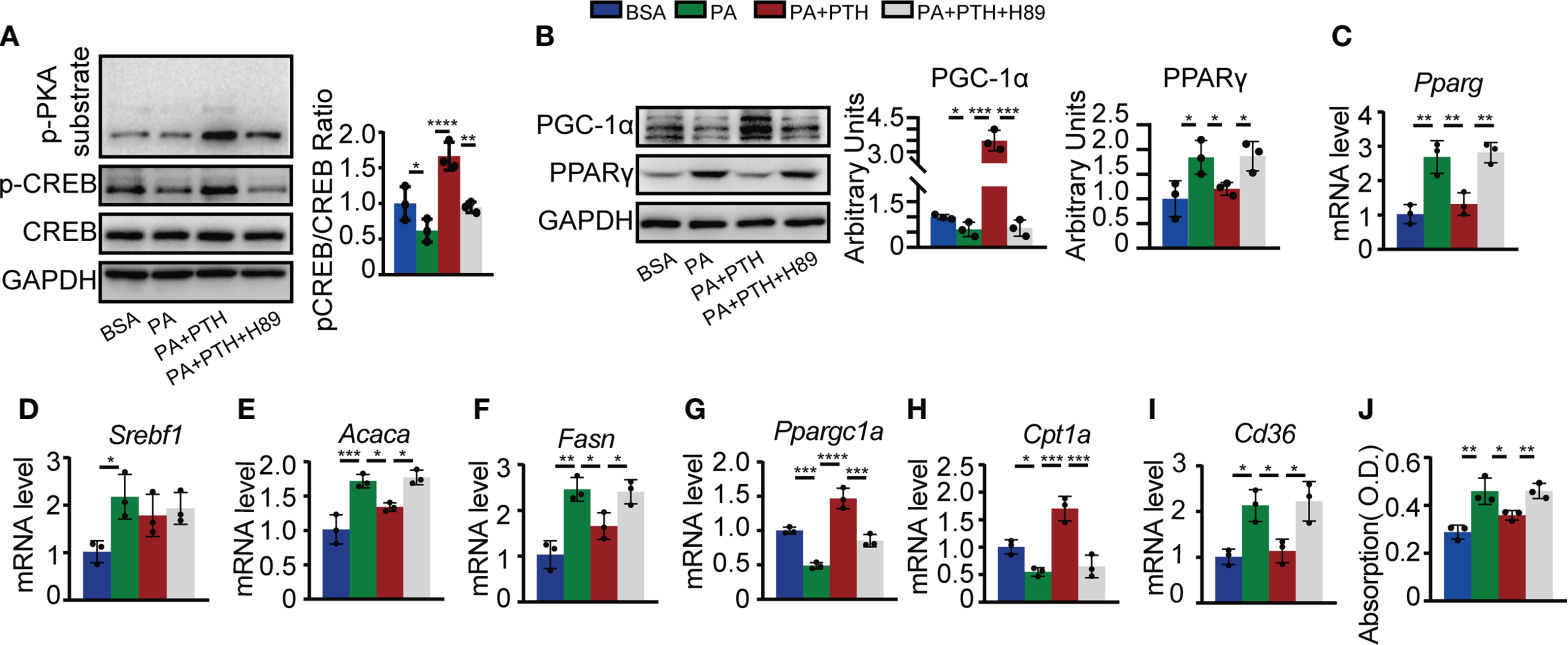
PTH regulated lipid metabolism *via* the cAMP/PKA/CREB pathway. Hepa1-6 cells were pretreated with 10 μM H89 for 1h, and then 0.3 mM PA and 10 nM PTH for 24h. **(A)** The protein levels of p-PKA substrates, p-CREB, CREB, and GAPDH. **(B)** The protein levels of PGC-1α, PPARγ, and GADPH. **(C–I)** The mRNA levels of *Pparg*, *Srebf1*, *Acaca*, *Fasn*, *Ppargc1a*, *Cpt1a*, and *Cd36*. **(J)** Quantitative assessment of intracellular lipid content in PA-treated Hepa1-6 cells. Data are shown as mean ± SD, the cell experiments were repeated three times. *P < 0.05; **P < 0.01; ***P < 0.001; ****P < 0.0001 by one-way ANOVA.

Canonically, PTH and PTHrP binding to PTH1R could elevate the cAMP level and thus activate downstream signaling pathways such as PKA/CREB pathway (11, 40, 41). The PKA/CREB pathway is known to be involved in hepatic lipid metabolism by regulating PGC-1α and PPARγ (42, 43). To elucidate whether PTH promoted hepatic lipid metabolism *via* PKA/CREB pathway, we pretreated Hepa1-6 cells with PKA inhibitor H89 or DMSO as a control and then incubated Hepa1-6 cells with PA and PTH. As shown in **Figure 6A**, PA-induced decrease in levels of p-PKA substrate and p-CREB were reversed by PTH treatment, while pretreatment with H89 abolished these effects (**Figure 6A**). The protein levels of PGC-1α and PPARγ elevated by PTH treatment were blocked in the presence of H89 (**Figure 6B**). Furthermore, H89 suppressed the *Ppargc1a*, and *Cpt1a* levels for lipid β-oxidation, upregulated the *Pparg*, *Acaca*, and *Fasn* levels for *de novo* lipogenesis, and *Cd36* level for lipid uptake, compared with those of PTH-treated Hepa1-6 cells (**Figures 6C** **–I**). Consistently, the effects of PTH on reducing intracellular lipid accumulation were also diminished by H89 (**Figure 6J**). Collectively, we concluded that PTH activates the PKA/CREB pathway to regulate lipid homeostasis in PA-treated Hepa1-6 cells.

## Discussion

Non-alcoholic fatty liver disease is becoming a global concern and lacks licensed therapeutics at present (2, 4–7). As James Black famously stated, “the best way to discover a new drug is to start with an old one” (44). In the current study, we explored the novel therapeutic effects of PTH on liver steatosis. PTH was the only FDA-approved anabolic treatment for osteoporosis. Despite the classical effects on bone and kidney to regulate serum calcium-phosphate homeostasis, emerging evidence has indicated an important role of PTH in the lipid metabolic process (11–15). Our findings showed that intermittent administration of PTH reduced body weight gain and fat mass, and alleviated hepatic steatosis in both DIO mice and mid-life db/db mice. Additionally, hepatic lipid metabolism-related genes were markedly affected by PTH treatment. *In vitro*, PTH inhibited PA-induced intracellular lipid accumulation in Hepa1-6 cells. These results suggested a potential therapeutic role of PTH on hepatic steatosis.

Notably, excess PTH level was correlated with increased serum free fatty acid due to lipolytic action of adipose tissue (9, 10), which may flux into the liver contributing to hepatic steatosis. Furtherly, circulation tumor-derived PTHrP, which resembles PTH, was linked to cancer cachexia (11). In our study, however, intermittent administration of PTH did not affect the body weight, liver weight, or liver histology in mice fed with NCD. Moreover, the gross phenotype and weights of brown adipose tissues showed no obvious changes after PTH treatment in DIO mice (**Supplementary Figures 1B, C**), while the weights of sWAT and eWAT decreased in both DIO mice and db/db mice. We considered that our low dose of PTH was insufficient to provoke excessive adipocyte lipolysis in the presence of insulin *in vivo (* 12), and the decreased fat weight could be partly attributed to the improved hepatic lipid metabolism.

PTH binds to PTH1R and activates the downstream signaling pathway to exert its function (40). Previous studies and our results proved that PTH1R is constitutively expressed in hepatic cells (12, 38, 39). We found that the inhibitory effect of PTH on PA-induced lipid accumulation was abolished by PTH1R knockdown in Hepa1-6 cells, demonstrating a direct and PTH1R-dependent effect of PTH on hepatocytes. By utilizing PKA inhibitor H89, we further demonstrated that PTH substantially affected the expression of a set of genes related to *de novo* lipogenesis, uptake, and β-oxidation of lipid *via* the cAMP/PKA/CREB pathway. Nevertheless, we cannot exclude possible contributions of other signaling cascades such as the protein kinase C pathway (12). Despite hepatic lipid metabolism, the involvement of cAMP/PKA/CREB pathway in hepatic gluconeogenesis indicates that PTH administration may counteract the effects of insulin (12, 45). But in fact, our results showed that intermittent administration of PTH did not disturb the insulin sensitivity in mice fed with NCD or HFD (**Supplementary Figures 1D, E**, **Supplementary Figures 2E, F**), which is consistent with other studies on PTH (15, 46, 47).

While PTH could benefit hepatic lipid metabolism, there remain some important issues to be addressed. In the clinical practice and our experiments, PTH is distributed all over the body through subcutaneous injection, and thus we could not exclude the side effects of PTH on the bone and kidney during the therapy. Furthermore, previous studies have raised a major concern about the increased risk of osteosarcoma (16). Together, we would prefer to suggest a new indication of PTH for patients suffering from both osteoporosis and NAFLD, and a targeted PTH delivery system for liver-specific delivery awaits further investigation.

## Data availability statement

The original contributions presented in the study are included in the article/**Supplementary Material**. Further inquiries can be directed to the corresponding authors.

## Ethics statement

The animal study was reviewed and approved by Laboratory Animal Ethics Committee at Central South University (2020SYDW0613).

## Author contributions

ZYX, JPW, and XF designed the experiments; XF carried out most of the experiments, generated data, and drafted the manuscript; YX, QG, HP, and HYZ helped to collect the samples; ZYX and JP W supervised the experiments, analyzed the data, proofread and revised the manuscript. All authors had access to the study data and had reviewed and approved the final manuscript.

## Funding

This work was supported by grants from the National Natural Science Foundation of China (NO. 82000848, 81900810, 82170866), the Natural Science Foundation of Hunan Province, China (NO. 2019JJ40262).

## Conflict of interest

The authors declare that the research was conducted in the absence of any commercial or financial relationships that could be construed as a potential conflict of interest.

## Publisher’s note

All claims expressed in this article are solely those of the authors and do not necessarily represent those of their affiliated organizations, or those of the publisher, the editors and the reviewers. Any product that may be evaluated in this article, or claim that may be made by its manufacturer, is not guaranteed or endorsed by the publisher.

## Supplementary material

Supplementary Figure 1 Effects of PTH on DIO mice. Eight-week-old male C57/BL6 mice were fed with HFD and subcutaneously injected with PTH (40 μg/kg daily) or PBS for 16 weeks. **(A, B)** Gross pictures of the whole body, sWAT, eWAT, and BAT (Bar scale = 1cm). **(C)** BAT weight. **(D, E)** Insulin tolerance test **(D)** and AUC **(E)**. Data are shown as mean ± SD (n=5/group).

Supplementary Figure 2 Effects of PTH on normal chow diet-fed mice. Eight-week-old male C57/BL6 mice were fed with a normal chow diet and subcutaneously injected with PTH (40 μg/kg daily) or PBS for 2 months. **(A)** Body weight curve. **(B)** Daily food intake. **(C)** Liver weight. **(D)** H&E staining of liver tissues (Scale bar = 250 μm). **(E, F)** Insulin tolerance test **(E)** and AUC **(F)**. Data are shown as mean ± SD (n=5/group).

Supplementary Figure 3 Acute fasting induced hepatic steatosis. Physiological parameters of twelve-week-old C57/BL6 mice after 24 h fasting. **(A)** The H&E staining and Oil Red O staining of liver tissues (Scale bar = 100 μm). **(B, C)** The body weights and liver weights of fed mice and fasted mice. **(D, E)** The hepatic TG and TC levels. **(F, G)** The serum TG and TC levels. **(H, J)** The serum ALT and AST levels. Data are shown as mean ± SD (n=4/group). **P < 0.01; ***P < 0.001 by two-tailed Student’s t-test.
