# Supplementary figures and images for: Parathyroid hormone alleviates non-alcoholic liver steatosis via activating the hepatic cAMP/PKA/CREB pathway
Source: Front Endocrinol (Lausanne). 2022 Aug 17;13:899731. doi: 10.3389/fendo.2022.899731 (PMC9428460; doi:10.3389/fendo.2022.899731)

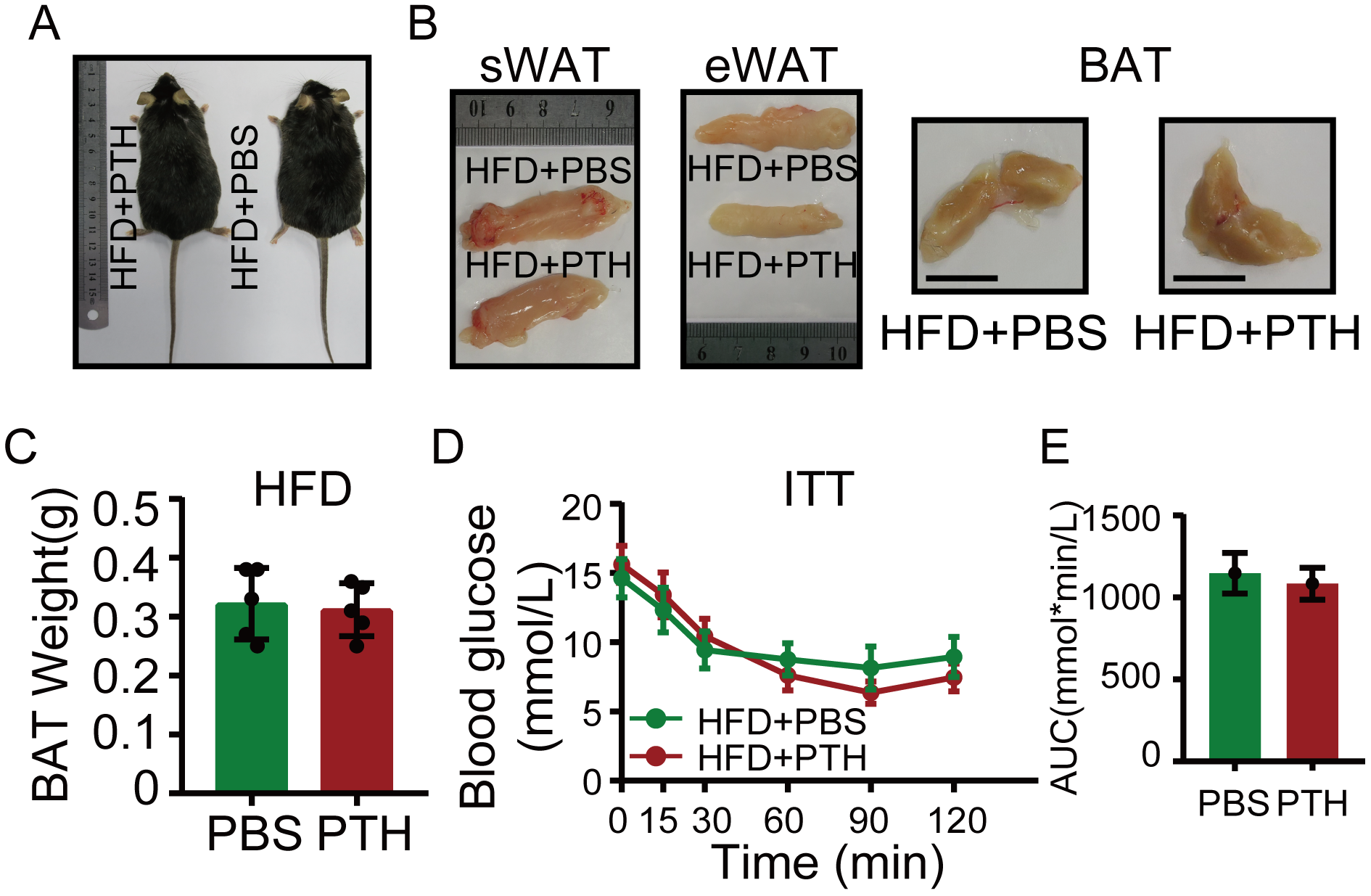

Supplement: Supplementary Figure 1 — Effects of PTH on DIO mice. Eight-week-old male C57/BL6 mice were fed with HFD and subcutaneously injected with PTH (40 μg/kg daily) or PBS for 16 weeks. (A, B) Gross pictures of the whole body, sWAT, eWAT, and BAT (Bar scale = 1cm). (C) BAT weight. (D, E) Insulin tolerance test (D) and AUC (E). Data are shown as mean ± SD (n=5/group). [file Image_1.tif]

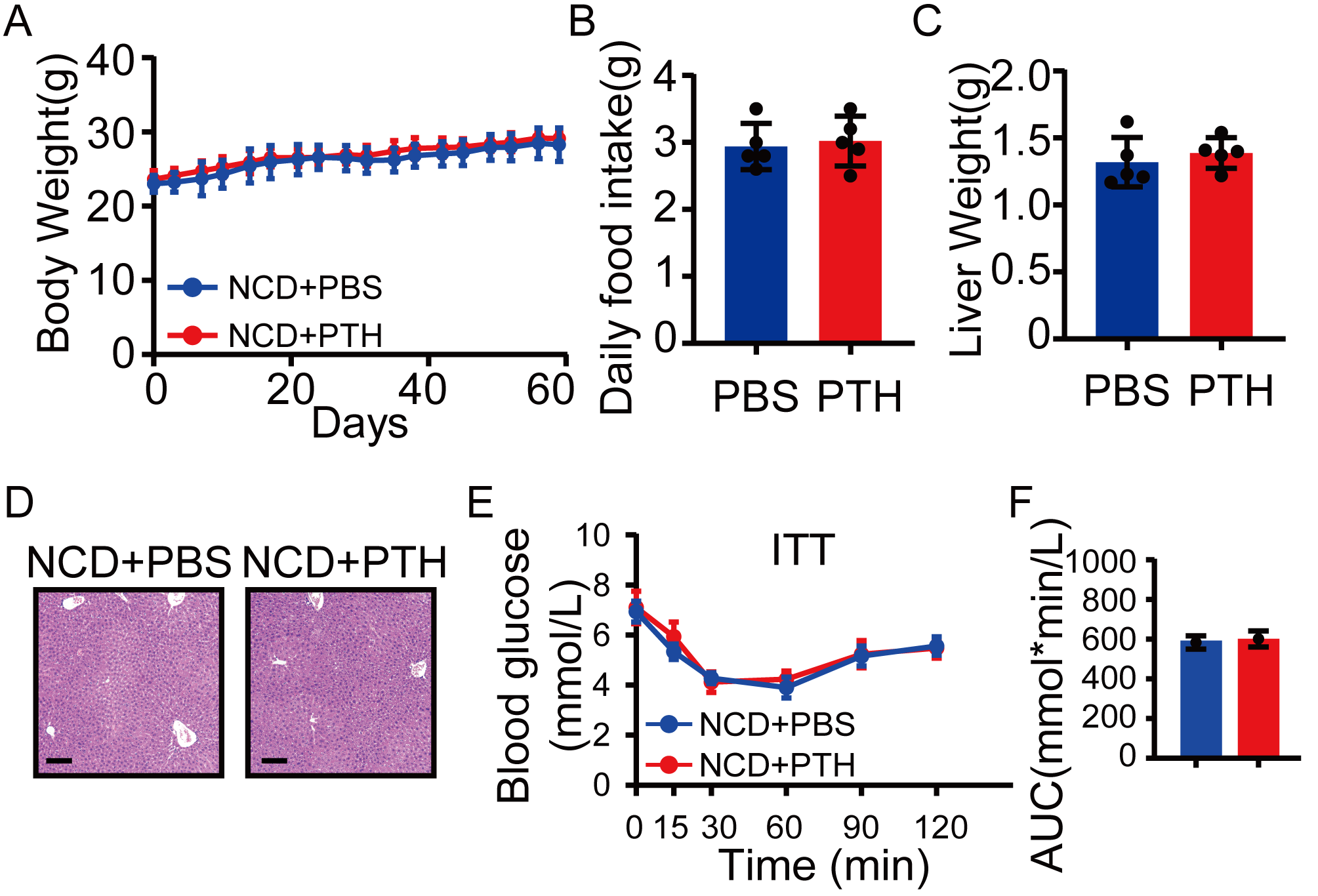

Supplement: Supplementary Figure 2 — Effects of PTH on normal chow diet-fed mice. Eight-week-old male C57/BL6 mice were fed with a normal chow diet and subcutaneously injected with PTH (40 μg/kg daily) or PBS for 2 months. (A) Body weight curve. (B) Daily food intake. (C) Liver weight. (D) H&E staining of liver tissues (Scale bar = 250 μm). (E, F) Insulin tolerance test (E) and AUC (F). Data are shown as mean ± SD (n=5/group). [file Image_2.tif]

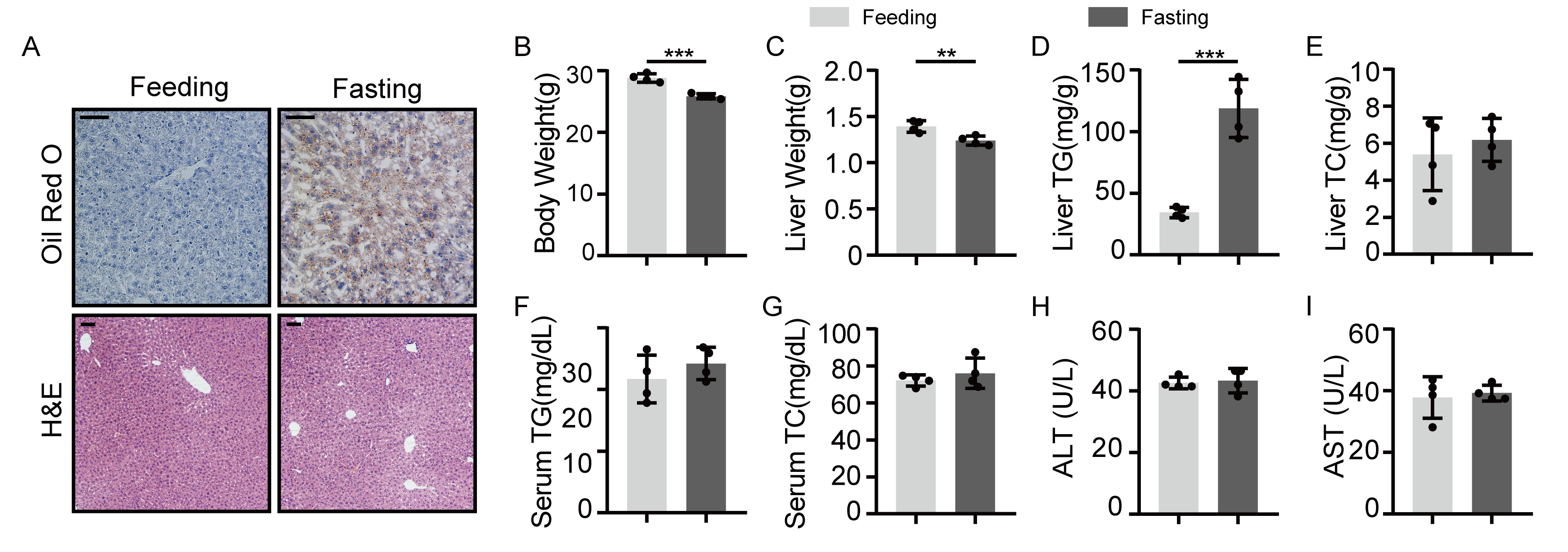

Supplement: Supplementary Figure 3 — Acute fasting induced hepatic steatosis. Physiological parameters of twelve-week-old C57/BL6 mice after 24 h fasting. (A) The H&E staining and Oil Red O staining of liver tissues (Scale bar = 100 μm). (B, C) The body weights and liver weights of fed mice and fasted mice. (D, E) The hepatic TG and TC levels. (F, G) The serum TG and TC levels. (H, J) The serum ALT and AST levels. Data are shown as mean ± SD (n=4/group). **P < 0.01; ***P < 0.001 by two-tailed Student’s t-test. [file Image_3.tif]
