# Supplementary material for: Parathyroid hormone alleviates non-alcoholic liver steatosis via activating the hepatic cAMP/PKA/CREB pathway
Source: Front Endocrinol (Lausanne). 2022 Aug 17;13:899731. doi: 10.3389/fendo.2022.899731 (PMC9428460; doi:10.3389/fendo.2022.899731)
Supplement: Supplementary file 4 [file Table_1.docx]

**Supplementary Table 1**

Nucleotide sequences of primers used for qRT-PCR

| *Gapdh* | F: AGGTCGGTGTGAACGGATTTG |
| --- | --- |
|  | R: TGTAGACCATGTAGTTGAGGTCA |
| *Pth1r* | F: CAGGCGCAATGTGACAAGC |
|  | R: TTTCCCGGTGCCTTCTCTTTC |
| *Pparg* | F: TCGCTGATGCACTGCCTATG |
|  | R: GAGAGGTCCACAGAGCTGATT |
| *Srebf1* | F: TGACCCGGCTATTCCGTGA |
|  | R: CTGGGCTGAGCAATACAGTTC |
| *Acaca* | F: ATGGGCGGAATGGTCTCTTTC |
|  | R: TGGGGACCTTGTCTTCATCAT |
| *Fasn* | F: GGAGGTGGTGATAGCCGGTAT |
|  | R: TGGGTAATCCATAGAGCCCAG |
| *Ppargc1a* | F: TATGGAGTGACATAGAGTGTGCT |
|  | R: CCACTTCAATCCACCCAGAAAG |
| *Cpt1a* | F: CTCCGCCTGAGCCATGAAG |
|  | R: CACCAGTGATGATGCCATTCT |
| *Cd36* | F: AGATGACGTGGCAAAGAACAG |
|  | R: CCTTGGCTAGATAACGAACTCTG |

(F, forward primer; R, reverse primer.)
